# Psoriasis with onychomycosis in a diabetic patient

**DOI:** 10.11604/pamj.2021.40.72.26025

**Published:** 2021-10-01

**Authors:** Surya Besant Natarajan, Krishna Prasanth Baalann

**Affiliations:** 1 Department of Community Medicine, Sree Balaji Medical College and Hospital, Bharath University, Chennai, India

**Keywords:** Onychomycosis, psoriasis, diabetes

## Image in medicine

Global prevalence of psoriasis ranges between 0.09% to 11.43%. Psoriasis is basically a defective inflammatory response. Dry flakes of silvery white skin scales in psoriasis results from the excessive proliferation of skin. Incessant irritation brought about by psoriasis is the most significant and huge contributing component for the expanded diabetes hazard and different other metabolic disorders. A 45-year-old man presented with complaints of yellowish discoloration of nails on both hands with difficulty to perform day to day activities. Patient a known case of psoriasis for the past 7 years and was on topical medication for the same. Examinations revealed yellowish discoloration of fingernails, thickening of nails, with separation of nail from nail bed. A deep ulceration of size 1 x 2 cm over the distal phalanx is noticed. Lab investigations revealed poor glycemic control and on direct microscopy with 20% potassium hydroxide (KOH) and culture revealed onychomycosis. Patient was started on systemic anti-fungals medication and simultaneous treatment for achieving glycemic control.

**Figure 1:**
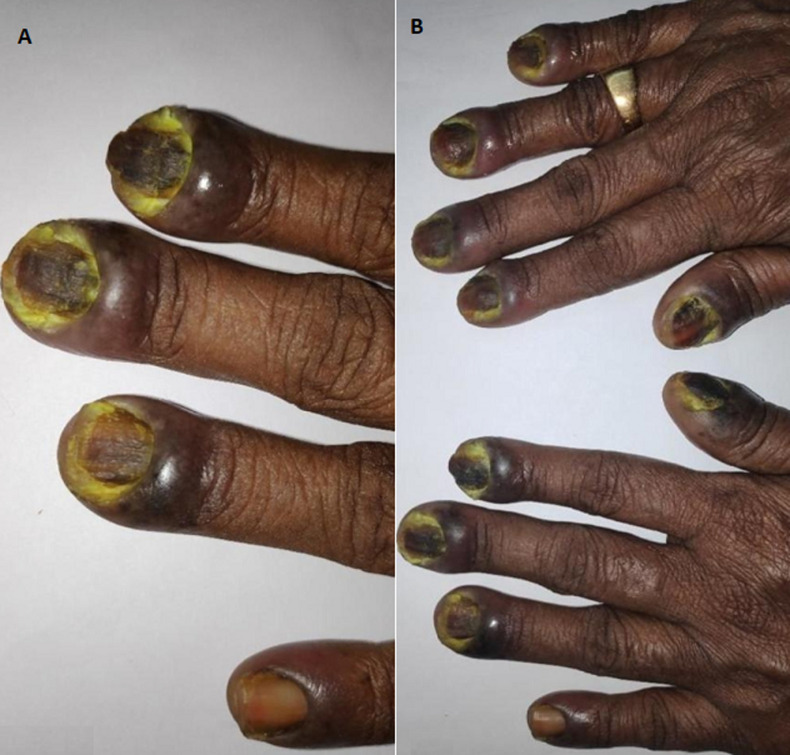
(A,B) ulcerations due to onychomycosis

